# Bridging the Beam Gap: Expanding Global Access to Stereotactic Radiosurgery Through Policy and Innovation

**DOI:** 10.7759/cureus.89268

**Published:** 2025-08-02

**Authors:** Quang Dai La, Sreyansh Rishabh, Shanmukh Bachhu, Tatsuya Tanaka, Marc Faltas, Kerol Faltas, Nehal Revuri, Aiman Baloch, Han B La, Francis Pryor, Eduardo E Lovo

**Affiliations:** 1 Medicine, The Innovative STEMagazine, College Station, USA; 2 Biology, Texas A&M University, College Station, USA; 3 Kinesiology, Pennsylvania State University Harrisburg, Middletown, USA; 4 Civil Engineering, University of California - Berkeley, Berkeley, USA; 5 Engineering, The Innovative STEMagazine, College Station, USA; 6 Neurosurgery, International University of Health and Welfare Narita Hospital, Narita, JPN; 7 Medicine, Rowan-Virtua School of Osteopathic Medicine, Stratford, USA; 8 Medicine, University of Pennsylvania Perelman School of Medicine, Philadelphia, USA; 9 Neurological Surgery, The Innovative STEMagazine, College Station, USA; 10 Medicine, Mekran Medical College, Turbat, PAK; 11 Medicine, Texas A&M University College of Medicine, College Station, USA; 12 Medicine, Lake Erie College of Osteopathic Medicine, Erie, USA; 13 Neurosurgery-Gamma Knife Program, International Cancer Center, Diagnostic Hospital, San Salvador, SLV

**Keywords:** gamma knife, global health equity, global neuro-oncology, health policy and innovation, linac-based radiosurgery, low- and middle-income countries (lmics), radiosurgery access, radiotherapy in resource-limited settings, stereotactic radiosurgery (srs), workforce development in neurosurgery

## Abstract

Stereotactic radiosurgery (SRS), a noninvasive technique that delivers a high dose of ionizing radiation to a precisely defined focal target volume, is foundational to modern neuro-oncology and functional neurosurgery. SRS provides highly accurate, noninvasive treatment for a range of intracranial conditions, including malignant and benign tumors, vascular malformations such as arteriovenous malformations (AVMs), and movement or functional disorders like trigeminal neuralgia. Despite a well-documented safety record and demonstrable efficacy, significant disparities in accessibility persist across global, geographic, and socioeconomic lines. This editorial aims to explore ways in which health policy innovation, international collaboration, and investment into mobile or modular radiosurgery platforms can help bridge gaps and promote access to SRS interventions for underserved populations. Addressing these disparities requires a thoughtful discourse on outcome equity and the ethical imperative to ensure universal access to neurosurgical care.

## Editorial

**Figure 1:**
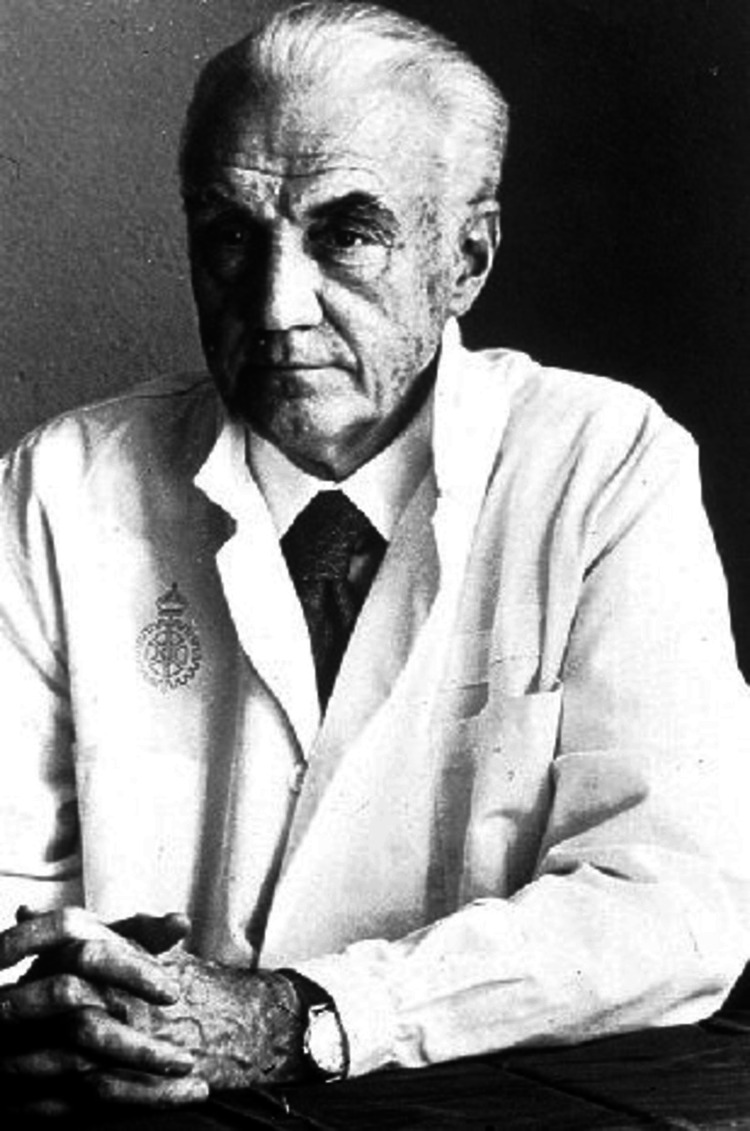
Lars Leksell (1907–1986) was a Swedish physician and Professor of Neurosurgery at the Karolinska Institute in Stockholm, Sweden, renowned for inventing radiosurgery. Image reproduced from Saceleanu et al. (2020) [3] under the Creative Commons Attribution-NonCommercial-ShareAlike 4.0 International Public License.

Stereotactic radiosurgery (SRS) was developed by Dr. Lars Leksell (Figure 1) in 1951 and has transformed the therapeutic landscape of intracranial pathologies such as arteriovenous malformations, vestibular schwannomas, and brain metastases by offering a noninvasive treatment modality that significantly reduced the need for open craniotomy in selected cases [1-3]. In high-income countries, SRS is a safer option with even less time in recovery than open surgery [4].

**Figure 2:**
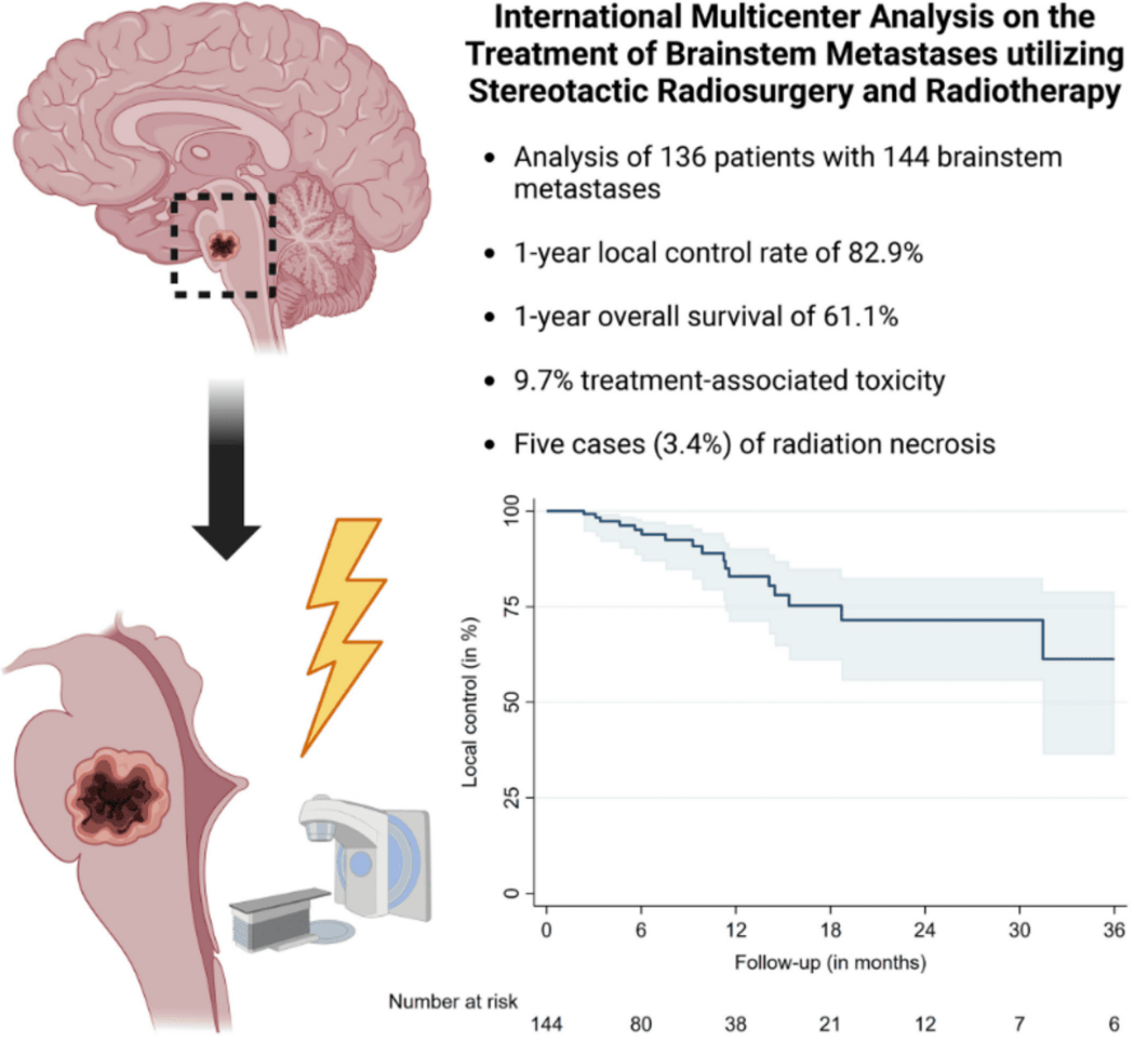
Graphical abstract summarizing an international multicenter study showing that stereotactic radiosurgery and fractionated stereotactic radiotherapy offer effective and safe long-term control of brainstem metastases. Image reproduced from Ehret et al. 2024 [5] under the terms of the Creative Commons Attribution-NonCommercial License.

The clinical utility and flexibility of SRS have expanded exponentially in the last decade, and randomized controlled trials support the use of SRS to treat brain metastases (Figure 2) [5]. The *Lancet's* JLGK0901 prospective observational study set forth evidence that SRS provides comparable outcomes to whole-brain radiation therapy (WBRT) in patients with up to 5-10 intracranial lesions [6,7]. In the JLGK0901 study (n = 1,194), SRS-alone for 5-10 metastases was shown to provide noninferior overall survival and equivalent neurocognitive preservation compared to SRS-alone for treatment of 2-4 metastases [6,8]. Importantly, it has been demonstrated through trials like the *Journal of the American Medical Association's* (JAMA’s) N0574 randomized study that patients with SRS (1-3 metastases) showed significantly less cognitive decline at three-month follow-up, 63% vs. 92% with SRS + WBRT, and also improved quality of life, without any compromise for survival [9]. SRS is fast solidifying itself at the forefront of options in the noninvasive neuro-oncology community.

However, SRS remains largely inaccessible in low- and middle-income countries (LMICs). For example, only about half of African countries had any megavoltage radiotherapy unit in 2020, with equipment concentrated in Egypt and South Africa [10]. An International Atomic Energy Agency (IAEA) evaluation of 54 African countries in March 2020 reported that only 52% had teletherapy units, and none were able to meet the projected radiotherapy load in 2030 (an increase from 0.84 million to more than 1.5 million annual cancer cases) [10]. Given the long wait periods for conventional radiotherapy, SRS is often viewed as a luxury; time is a scarce resource in these settings.

Apart from perceptions of luxury, a few other constraints hinder SRS implementation. First, SRS platforms such as Gamma Knife or dedicated linear accelerator (LINAC)-based systems involve significant upfront and ongoing maintenance costs that are prohibitive, especially in comparison to, for example, basic teletherapy units [10,11]. Second, health systems in LMICs often lack health technology assessment to assess technology use and implementation, and there is uncertainty about whether there is a cost-effective return on investment for SRS versus competing priorities like infectious disease control or maternal health. Iqbal et al. noted that there is currently limited and context-dependent evidence on cost-effectiveness, and there was clear uncertainty about SRS implementation compared to surgical resection in lower-middle-income countries like Vietnam, even with strong supportive evidence in high-income countries like Germany [11]. Third, few trained multidisciplinary teams (the implementation of equipment requires neurosurgeons, medical physicists, dosimetrists, and radiation oncologists) indicated to have "serious" issues with workforce shortages and workforce training infrastructure in LMIC neurosurgery systems [11]. Finally, fragile infrastructure increases equipment downtime: technical failure rates in LINAC systems are significantly higher in LMIC environments (e.g., Nigeria, Botswana, and Indonesia), with long repair durations, particularly for subsystems like multi-leaf collimators (MLCs), reducing operational efficiency and raising operating costs [12-14].

Over 70% of cancer patients in LMICs do not receive timely radiotherapy [12,13]. As an additional example, in sub-Saharan Africa, more than 50% of countries are without any radiotherapy infrastructure at all, which leads to individual countries having less than 0.2 megavoltage units per million population [14]. In Latin America and the Caribbean, only three out of 40 countries meet the IAEA machine-density targets, and even in countries that do have facilities (e.g., Brazil and Mexico), service provision was highly centralized for those who can access it [15]. The issue is similar for Southeast and South Asia; even though an enormous population bears the burden of cervical cancer, only around 40% of eligible women receive radiotherapy [16]. In many countries, there are only 50 or fewer machines across the entire country, and often all are located in urban environments, meaning clusters of services [16].

There are also technical limitations: for example, in LMICs, LINACs are down for long periods of time (on average ~14 days per failure), with MLCs causing most of the downtime [13,17]. A comparative assessment indicated that LINAC subsystem failure rates, especially vacuum pumps in LMICs, are more than twice that of high-income countries (HICs), with long-duration (>1 hours) faults being nearly 75% of the total downtime [17]. While the downtime data applies to general radiotherapy LINACs rather than SRS-specific systems, it highlights the maintenance challenges that could similarly affect the high-precision systems used in dedicated SRS systems. 

Even when they are available, LINACs are often overwhelmed by radiotherapy demands. The added time required for SRS, including quality assurance, patient treatment, and specialized training, makes it difficult to implement in already strained systems. Dedicated SRS systems like the Gamma Knife bypass these constraints and have been successfully scaled in resource-limited settings. Alternatives such as compact LINAC systems and head-based cobalt-60 platforms (e.g., Gamma Knife Icon, Elekta AB, Stockholm, Sweden) offer a high degree of accuracy while requiring less infrastructure to install and maintain [18]. Prototype mobile SRS devices and hub-and-spoke models have shown potential in resource-limited environments [19].

Workforce development is crucial. Organizations have been able to conduct longitudinal remote SBRT/SRS training for clinicians in Latin America with success, illustrating significant improvement in knowledge, confidence, and competency across various domains through a weekly telehealth approach and case-based learning [20,21]. We need to take such initiatives to scale-using multi-language curricula, virtual simulators, and sustainable funding-to strengthen permanent networks and capacity development in the underserved regions.

Beyond investment in technology and workforce, interventions in policy are necessary to realize and enable the sustainability of SRS in LMICs. National cancer control plans that explicitly include SRS should be developed in LMICs. Funding should then be provided from the public purse through a tax-based system or social insurance scheme to reduce the impact of high-precision treatment on out-of-pocket payments by patients when utilizing an SRS service [21,22]. In parallel, efforts should be made to establish regulatory policies and independent regulatory authorities for radiation safety to oversee safe procurement, siting, operation, and maintenance of SRS equipment, including licensing, quality assurance, and compliance [23,24]. Incentivizing the development of SRS capacity in underserved areas in LMICs by way of providing financial incentives via funding or tax breaks for private providers and requiring SRS service programs in rural or low-income locations for government licensure or funding [25]. For a sustainable environment for SRS, policy must incorporate transparent multi-stakeholder planning explicitly; involve importantly ministries of health/finance, local champions, civil society, and NGOs, and global implementation agencies; and importantly employ some version of a readiness-assessment framework such as the radiotherapy essential skills and equipment for awareness (RESEA) readiness-assessment framework [23,24]. For planning and sustaining radiotherapy services, the RESEA framework provides government-led appraisal involving four domains: commitment, cooperation, capacity, and catalyst to form a sustainable service. Integrating all of the above policy elements into the governance creates an enabling environment that can facilitate not only the scoping and rollout of SRS but also the experience of safety, equity, and impact on global cancer care.

SRS offers a non-invasive alternative that could have significant value in settings with limited access to conventional neurosurgery. However, its adoption requires careful consideration of infrastructure and workforce limitations. Expanding dedicated SRS systems to underserved populations is not only a matter of technological advancement in LMICs but also an ethical imperative and long overdue in addressing profound inequity in global access to neurosurgical care and healthcare. The minimally invasive nature and strong safety profile of SRS make it a compelling option for underdeveloped areas with limited availability of neurosurgery care. With a coordinated global effort, the promise of SRS can be fully realized as a step towards global health equity outcomes.

## References

[1] Leksell L (1951). The stereotaxic method and radiosurgery of the brain. Acta Chir Scand.

[2] Leksell DG (1987). Stereotactic radiosurgery. Present status and future trends. Neurol Res.

[3] Săceleanu MV, Mohan AG, Marinescu AA, Ciurea AV (2020). 100 Years since the birth of Ladislau Steiner. Creativity of neurosurgery. Rom J Morphol Embryol.

[4] Sheehan JP, Yen CP, Lee CC, Loeffler JS (2014). Cranial stereotactic radiosurgery: current status of the initial paradigm shifter. J Clin Oncol.

[5] Ehret F, Rueß D, Blanck O (2024). Stereotactic radiosurgery and radiotherapy for brainstem metastases: an international multicenter analysis. Int J Cancer.

[6] Yamamoto M, Serizawa T, Shuto T (2014). Stereotactic radiosurgery for patients with multiple brain metastases (JLGK0901): a multi-institutional prospective observational study. Lancet Oncol.

[7] Gondi V, Bauman G, Bradfield L (2022). Radiation therapy for brain metastases: an ASTRO clinical practice guideline. Pract Radiat Oncol.

[8] Yamamoto M, Serizawa T, Higuchi Y (2017). A multi-institutional prospective observational study of stereotactic radiosurgery for patients with multiple brain metastases (JLGK0901 study update): irradiation-related complications and long-term maintenance of mini-mental state examination scores. Int J Radiat Oncol Biol Phys.

[9] Brown PD, Jaeckle K, Ballman KV (2016). Effect of radiosurgery alone vs radiosurgery with whole brain radiation therapy on cognitive function in patients with 1 to 3 brain metastases: a randomized clinical trial. JAMA.

[10] Elmore SN, Polo A, Bourque JM (2021). Radiotherapy resources in Africa: an International Atomic Energy Agency update and analysis of projected needs. Lancet Oncol.

[11] Iqbal J, Naseem A, Bashir MA, Yangi K, Bozkurt I, Chaurasia B (2025). Global perspective of neurosurgery practice in lower middle-income countries: challenges, opportunities, and the path forward. Ann Med Surg (Lond).

[12] Grover S, Xu MJ, Yeager A (2014). A systematic review of radiotherapy capacity in low- and middle-income countries. Front Oncol.

[13] Peiris GS, Pawiro SA, Kasim MF, Sheehy SL (2025). Failure modes and downtime of radiotherapy linear accelerators and multi‑leaf collimators in Indonesia (Preprint). arXiv.

[14] Bishr MK, Zaghloul MS (2018). Radiation therapy availability in Africa and Latin America: two models of low and middle income countries. Int J Radiat Oncol Biol Phys.

[15] (2025). Radiotherapy inaccessibility in lung cancer care: an ongoing crisis. https://www.ilcn.org/radiotherapy-inaccessibility-in-lung-cancer-care-an-ongoing-crisis/.

[16] Bhatia R, Lichter KE, Gurram L, MacDuffie E, Lombe D, Sarria GR, Grover S (2022). The state of gynecologic radiation therapy in low- and middle-income countries. Int J Gynecol Cancer.

[17] Wroe LM, Ige TA, Asogwa OC (2020). Comparative analysis of radiotherapy linear accelerator downtime and failure modes in the UK, Nigeria and Botswana. Clin Oncol (R Coll Radiol).

[18] Meeks SL, Pukala J, Ramakrishna N, Willoughby TR, Bova FJ (2011). Radiosurgery technology development and use. J Radiosurg SBRT.

[19] Edwards DM, Kim MM (2024). Effective personalization of stereotactic radiosurgery for brain metastases in the modern era: opportunities for innovation. Cancer J.

[20] Sarria GR, Timmerman R, Hermansen M (2022). Longitudinal remote SBRT/SRS training in Latin America: a prospective cohort study. Front Oncol.

[21] (2019). Global, regional, and national burden of brain and other CNS cancer, 1990-2016: a systematic analysis for the Global Burden of Disease Study 2016. Lancet Neurol.

[22] Fezeu F, Awad AJ, Przybylowski CJ (2014). Access to stereotactic radiosurgery: identification of existing disparities and a modest proposal to reduce them. Cureus.

[23] Abdel-Wahab M, Coleman CN, Eriksen JG (2024). Addressing challenges in low-income and middle-income countries through novel radiotherapy research opportunities. Lancet Oncol.

[24] Donkor A, Luckett T, Aranda S, Vanderpuye V, Phillips JL (2021). Development of the 'REadiness SElf-assessment (RESEA) guide' to assist low and middle-income countries with establishing safe and sustainable radiotherapy services: a pragmatic sequential mixed qualitative methods project. BMC Health Serv Res.

[25] Flores JA, Lo CC, Tomagan JM (2024). Radiotherapy services in the Philippines: exploring geographical barriers to improve access to care. Lancet Reg Health West Pac.

